# Synthesis of Triazole Schiff’s Base Derivatives and Their Inhibitory Kinetics on Tyrosinase Activity

**DOI:** 10.1371/journal.pone.0138578

**Published:** 2015-09-30

**Authors:** Feng Yu, Yu-Long Jia, Hui-Fang Wang, Jing Zheng, Yi Cui, Xin-Yu Fang, Lin-Min Zhang, Qing-Xi Chen

**Affiliations:** 1 State Key Laboratory of Cellular Stress Biology, Key Laboratory of the Ministry of Education for Coastal and Wetland Ecosystems, School of Life Sciences, Xiamen University, Xiamen, 361005, China; 2 Key Laboratory for Chemical Biology of Fujian Province, Xiamen University, Xiamen, 361005, China; University of East Anglia, UNITED KINGDOM

## Abstract

In the present study, new Schiff’s base derivatives: (Z)-4-amino-5-(2-(3- fluorobenzylidene)hydrazinyl)-4H-1,2,4-triazole-3-thiol (Y_1_), (Z)-3-((2-(4-amino-5- mercapto-4H-1,2,4-triazol-3-yl)hydrazono)methyl)phenol (Y_2_), (Z)-2-((2-(4-amino-5- mercapto-4H-1,2,4-triazol-3-yl)hydrazono)methyl)phenol (Y_3_) and 3-((Z)-(2-(4- (((E)-3-hydroxybenzylidene)amino)-5-mercapto-4H-1,2,4-triazol-3-yl)hydrazono)methyl)phenol (Y_4_) were synthesized and their structures were characterized by LC-MS, IR and ^1^H NMR. The inhibitory effects of these compounds on tyrosinase activites were evaluated. Compounds Y_1_, Y_2_ and Y_3_ showed potent inhibitory effects with respective IC_50_ value of 12.5, 7.0 and 1.5 μM on the diphenolase activities. Moreover, the inhibition mechanisms were determined to be reversible and mixed types. Interactions of the compounds with tyrosinase were further analyzed by fluorescence quenching, copper interaction, and molecular simulation assays. The results together with the anti-tyrosinase activities data indicated that substitution on the second position of benzene ring showed superior ant-ityrosinase activities than that on third position, and that hydroxyl substitutes were better than fluorine substitutes. In addition, two benzene rings connecting to the triazole ring would produce larger steric hindrance, and affect the bonding between tyrosinase and inhibitors to decrease the inhibitory effects. The anti-tyrosinase effects of these compounds were in contrast to their antioxidant activities. In summary, this research will contribute to the development and design of antityrosinase agents.

## Introduction

Melanin existed in bacteria, fungi, plants and keratinocytes of skin and hair of animals, catalyzed by tyrosinase, made the surface coloring, which played an important role in protecting the skin and eye from ultraviolet radiation and preventing overheat of internal organization [1,2]. But overexpression of epidermal pigmentation may lead to some dermatological disorders, such as melasma, freckles, and senile lentigines [3].

Tyrosinase, a kind of multifunctional enzyme, mainly contributes to the melanin biosynthesis [4]. The enzyme could catalyze two distinct reactions involving the hydroxylation of monophenols and oxidation of diphenols to quinones [5]. The quinones could polymerize spontaneously to form macromolecular dark pigments or aggregate with amino acids and proteins to increase brown color of the pigment [6,7]. In addition, tyrosinase is involved in the process of insect molting, and fresh-keeping of fruits and vegetables [8–10]. In recent years, studies of tyrosinase mainly focus on pigment obstructive disease, melanoma, albino, early onset alzheimer's disease [11]. Therefore, it is of pressing need to acquire new tyrosinase inhibitors from different sources. Hydroquinone, kojic acid, azelaic acid, and arbutin as tyrosinase inhibitors have been applied in pharmaceuticals and cosmetics [12–15]. However, hydroquinone is prohibited for its irritation, mutagenesis and cytotoxic effects [16,17]. The use of kojic acid and arbutin are also limited because of their low efficacy in vivo, unsatisfactory formulation stability, and poor skin penetration [18]. Safe and efficient tyrosinase inhibitors will provid theoretical basis for the treatment of pigment disorders and enrich whitening cosmetics markets [19,20]. Mushroom tyrosinase as a mature model has been widely used in estimating of potential antityrosinase agents [21].

The copper ions in the active center of tyrosinase were the central part of catalytic activities of tyrosinase and it were found in the enzyme from different species [22,23]. So synthesis and screening of antityrosinase agents with copper chelating ability have become current research focus [24,25]. Heterocyclic compounds containing triazole ring have extensive biological activities such as antibacterial, antispasmodic, anti-inflammatory, especially a large number of derivatives have been synthesized as antibacterial drugs [26,27]. Because N and S atoms of the compounds played a key role in the coordination of metals at the active site of metalloprotein [28], they may have the ability to chelate the copper ions in active center of tyrosinase. So 1,2,4-triazole was widely used as mother nucleus to synthesize a series of special biological molecules, but few applications in the synthesis of tyrosinase inhibitor were reported. The structure of hydroxyl group on the benzene ring is similar to the enzyme substrate which can competitively inhibit the activity of enzyme. Therefore, using 4-Amino-3-hydrazino-5-mercapto-1,2,4-triazole (AHMZ, CAS No.1750-12- 5, the *IC*_50_ is 32.5 μM), a derivative of triazole, and benzaldehyde as potential moiety to make up a series of new Schiff's base molecules and explore their antityrosinase activities is the aims of our current work. The results could provide references for developing tyrosinase inhibitor as addition agent for use in fields of whitening cosmetics or fruit and vegetable preservation.

## Materials and Methods

### Materials

The tyrosinase, from mushroom, was bought from Sigma Chemical Co. (St. Louis, MO, USA) and the activity was 6680 U/mg. 4-Amino-3-hydrazino-5-mercapto-1, 2, 4-triazole, 3- hydroxy benzaldehyde, 2- hydroxy benzaldehyde and 3- fluorobenzene formaldehyde were obtained from Aladdin Industrial Co. (Shanghai, China). Other reagents were all analytical grade.

### Synthesis

**Fig 1:**
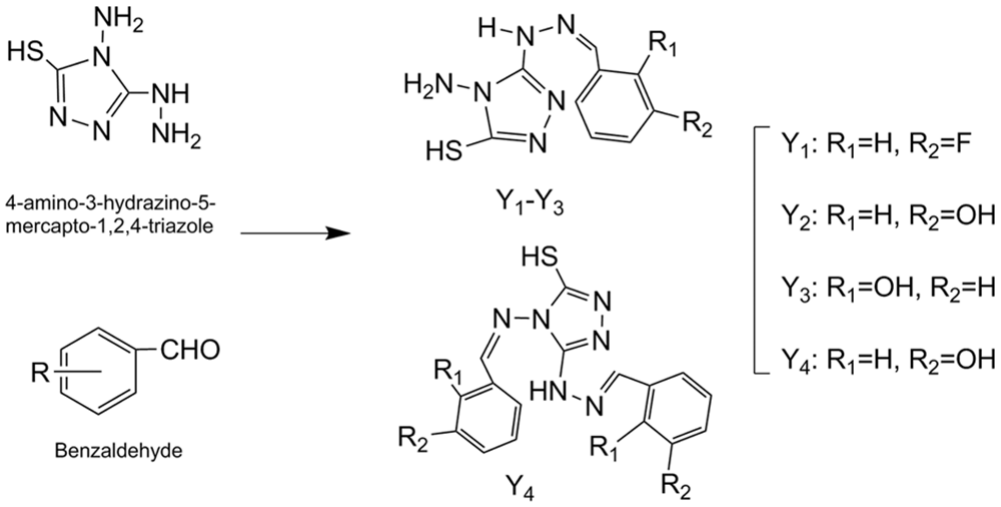
Synthetic processes of compound Y_1_, Y_2_, Y_3_ and Y_4_.

Schiff's base derivatives Y_1_, Y_2_, Y_3_ and Y_4_ have been synthesized by reactions between 4-Amino-3-hydrazino-5-mercapto-1,2,4-triazole and benzaldehyde derivatives in ethanol under reflux [29]. The products were filtrated and washed several times with ethanol and dried by suction filter. The synthetic procedures were described in Fig 1.

The structures of compounds Y_1_ to Y_4_ were verified by ^1^H NMR, IR and MS. The mass spectroscopy of compounds showed molecular ions peak [M+H]^+^.

### Enzyme assay

L-Tyr and L-DOPA were used as the substrate to test the monophenolase and diphenolase activities of tyrosinase, respectively [30]. The reaction system (3 ml) contained 1.1 mM L-Tyr or 0.5 mM L-DOPA, 25 mM PBS buffer (pH 6.8) and different concentrations of the inhbitors [31]. For monophenolase and diphenolase activities, the final concentration of tyrosinase was 33.3μg/ml and 6μg/ml, respectively. The increased optical density with the oxidation of the substrates at 475 nm (the absorption coefficient was 3700 M^−1^cm^−1^) within a certain time were measured by DU800 spectrophotometer [32]. IC_50_ was defined as the concentration of inhibitor inhibited 50% of the enzyme activity, which directly reflects the effect of the inhibitors. The Michaelis-Menten constant K_m_ of tyrosinase was determined by Lineweaver-Burk plot with different concentrations of L-DOPA as substrate and the inhibition constants K_I_ and K_IS_ were determined by the secondary plots of the apparent K_m_/V_m_ or 1/V_m_ versus the concentration of the inhibitors [33]. The inhibition mechanism was reflected by the plots of 1/v versus 1/[S] with various concentrations of mushroom tyrosinase [34].

### Fluorescence quenching experiments

Fluorescence quenching means the decrease of fluorescence intensity between the fluorescent molecules and solute molecules. This method was usually used to study the interaction between conformation of protein molecules and small molecules [35]. Cary Eclipse fluorescence spectrophotometer was used to record the fluorescence intensities with an excitation wavelength of 280 nm and emission slit widths of 5 nm [33]. Y_1_, Y_2_ and Y_3_ do not haven fluorescence phenomenon at the excitation wavelength. In this study, the inhibitor was added in 0.3 mg/ml tyrosinase solution to detect the fluorescence intensity changes and the final concentrations of inhibitor range from 10 to 70 μM.

### Copper interaction

The method to study the relationship between the copper ions and the compounds was similar to that described by Xiao-Xin Chen et al with slight modification [36, 37]. The reaction media include 0.5 mM PBS buffer, 33.3 μM inhibitor solutions and different concentrations of Cu^2+^. The DU800 spectrophotometer was used to record the spectra ranged from 300 nm to 450 nm 30 seconds after the addition of CuSO_4_.

### Antioxidant assay

ABTS free-radical scavenging assay was used to test the antioxidant capacity of compounds Y_1_ to Y_4_. The method can be found in many articles [38, 39]. 7 mM ABTS and 2.45 mM ammonium persulfate were blended and stored in a dark place 16 hours, and then diluted by 80% ethanol to obtain the working solution which gave an absorbance of 0.69 at 734 nm. The working solution and compounds were mixed and allowed to react for ten minutes, and then measured the absorbance at 734 nm (A_I_) was measured using spectrophotometer. L-ascorbic acid and 80% ethanol was used as a positive and blank control, respectively. The A_0_ value was the blank absorbance. The antioxidant rate (%) = (A_0_-A_I_)/A_0_ * 100%

### Molecular docking

The molecular docking technique allows better understanding of the potency of all compounds as inhibitors and the structure and activity relationship. We proceeded to examine the interactions of tyrosinase and compound Y_1_, Y_2_ and Y_3_. In this study, ChemDraw software was used to display the molecular models of the compounds and tyrosinase-inhibitors docking were demonstrated by molecular operation environment software (MOE). The polyphenol oxidase 3 (ppo3, PDB code: 2Y9W) without the structure of the exogenous protein, the PEG and water molecules was used as the protein model [40,41]. The bonding site of the enzyme in hydrophobic pocket nearby the copper ions was screened by software for the highest score. Before docking, the structure of tyrosinase molecule and ligand were energy minimized by MOE software.

## Results

### IR, ^1^H NMR and mass spectrometry

The products were white powders and dissolved in DMSO or water with alkali promoting. The followings were the data of IR, ^1^H NMR and LC-MS spectra of the compounds.

(Z)-4-amino-5-(2-(3-fluorobenzylidene)hydrazinyl)-4H-1,2,4-triazole-3-thiol (Y_1_): IR (S1 Fig) (KBr, νmax, cm-1) 3289, 3269(-NH_2_), 3197(-NH), 1648(C = N) ^1^H NMR (S2 Fig) (600 MHz, DMSO-d6) δ 10.84 (s, 1H), 8.32 (d, J = 2.3 Hz, 1H), 7.51–7.38 (m, 3H), 7.24–7.07 (m, 1H), 5.53 (d, J = 2.0 Hz, 2H), 5.28 (d, J = 2.1 Hz, 1H); LC-MS (m/z) (S3 Fig): observed, 253.35 [M+H]^+^; calculated, 252.27 [M]^+^.

(Z)-3-((2-(4-amino-5-mercapto-4H-1,2,4-triazol-3-yl)hydrazono)methyl)phenol (Y_2_): IR(S4 Fig) (KBr, νmax, cm-1) 3351(-OH), 3244, 3200(-NH_2_), 3153(-NH), 1651(C = N) ^1^H NMR (S5 Fig) (600 MHz, DMSO-d6) δ 12.94 (s, 1H), 10.60 (s, 1H), 9.53 (s, 1H), 8.23 (s, 1H), 7.21 (t, J = 7.8 Hz, 1H), 7.09 (t, J = 1.9 Hz, 1H), 6.99 (dt, J = 7.6, 1.2 Hz, 1H), 6.77 (ddd, J = 8.1, 2.6, 1.0 Hz, 1H), 5.50 (s, 2H); LC-MS (m/z) (S6 Fig): observed, 251.33 [M+H]^+^; calculated, 250.28 [M]^+^.

(Z)-2-((2-(4-amino-5-mercapto-4H-1,2,4-triazol-3-yl)hydrazono)methyl)phenol (Y_3_): IR (S7 Fig) (KBr, νmax, cm-1) 3448(-OH), 3283, 3247(-NH_2_), 3176(-NH), 1646(C = N) ^1^H NMR (S8 Fig) (600 MHz, DMSO-d6) δ 13.01 (s, 1H), 11.00 (s, 1H), 10.91 (s, 1H), 8.50 (d, J = 2.1 Hz, 1H), 7.48–7.37 (m, 1H), 7.24 (t, J = 7.7 Hz, 1H), 6.95–6.84 (m, 2H), 5.54 (d, J = 2.1 Hz, 2H); LC-MS (m/z) (S9 Fig): observed, 251.33 [M+H]^+^; calculated, 250.28 [M]+.

3-((Z)-(2-(4-(((E)-3-hydroxybenzylidene)amino)-5-mercapto-4H-1,2,4-triazol-3-yl)hydrazono)methyl)phenol (Y4): IR (S10 Fig) (KBr, νmax, cm-1) 3375(-OH), 3187, 3110(-NH2), 3020(-NH), 1637(C = N) 1H NMR (S11 Fig) (600 MHz, DMSO-d6) δ 10.68 (s, 1H), 9.98 (s, 1H), 9.84 (s, 1H), 9.57 (s, 1H), 8.25 (s, 1H), 7.45–7.28 (m, 3H), 7.22 (t, J = 7.8 Hz, 1H), 7.13 (t, J = 1.9 Hz, 1H), 7.03 (dt, J = 7.8, 1.7 Hz, 2H), 6.84–6.74 (m, 1H); LC-MS (m/z) (S12 Fig): observed, 355.42 [M+H]^+^; calculated, 354.39 [M]^+^.

### Effects of the compounds on mushroom tyrosinase

**Fig 2:**
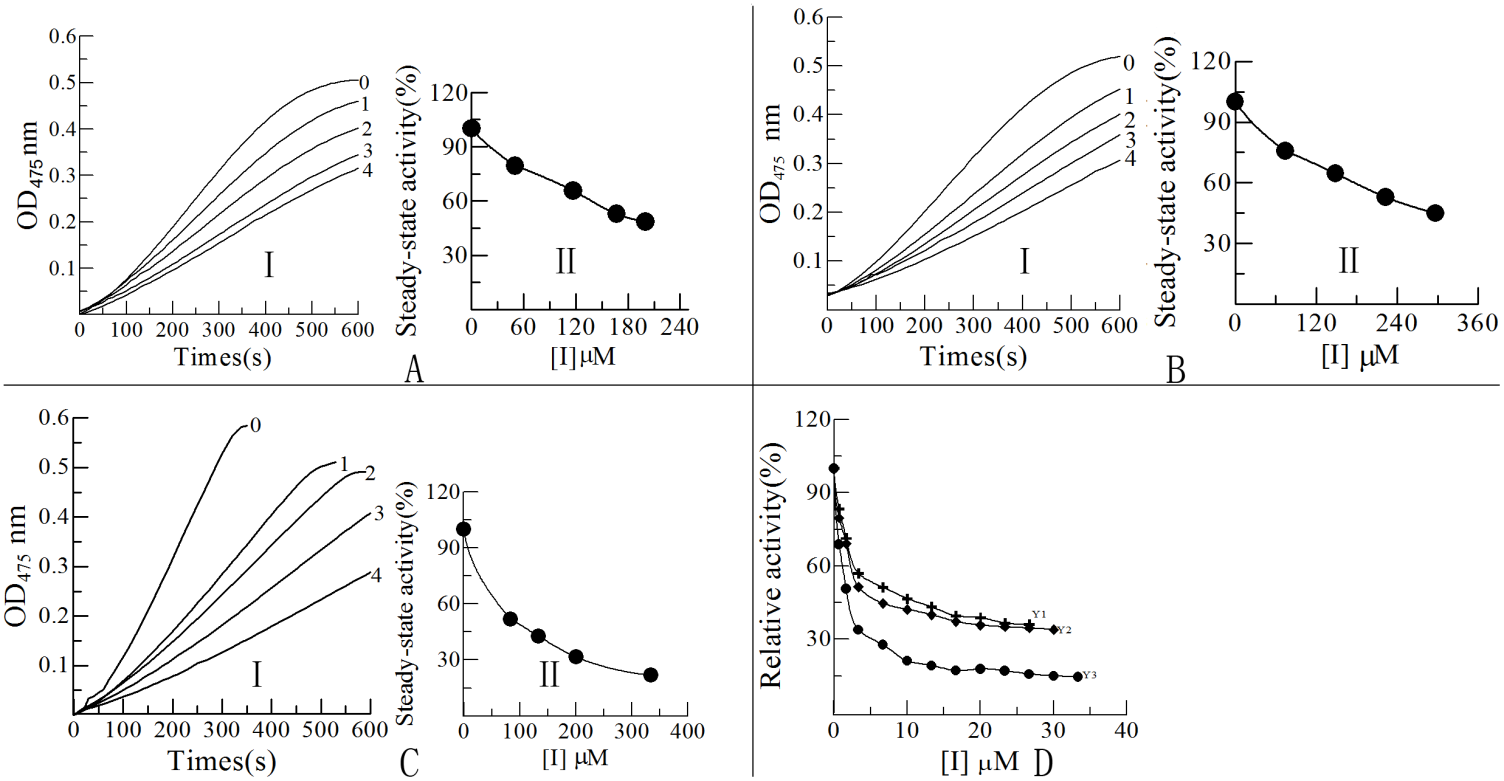
Determinations of the inhibitory activity on monophenolase and diphenolase. (**I**) The inhibitory effects on monophenolase activity of mushroom tyrosinase. (**II**) The inhibitory effects on steady-state activity of monophenolase. A, B and C represents the compound Y_1_, Y_2_ and Y_3_ respectively. (**D**) The inhibitory effects on diphenolase activity of mushroom tyrosinase.

L-DOPA as the diphenolase substrate of tyrosinase was used to screen the compounds with good antityrosinase activity. The results showed that compounds Y_1_ to Y_3_ had good inhibitory activity on diphenolase Fig 2D. Their IC_50_ values were determined to be 12.5, 7.0 and 1.5 μM, respectively. However, compound Y_4_ almost do not have antityrosinase activity. Then the inhibitory effects of the three compounds on monophenolase activity of tyrosinase were also determined. The results for the oxidation of the L-Tyr were shown in Fig 2AI, 2BI and 2CI, respectively.

In Fig 2, curves 0 to 4 expressed the monophenolase kinetics with different concentrations of inhibitors, and the activities of tyrosinase dropped significantly with the concentrations of the inhibitors increasing. After a certain period of time, the reaction slopes were constant and enzyme catalysis reached steady-state. The different slopes represented the stable activity of monophenolase, which reflected the oxidation rates of L-Tyr. The curves of Fig 2AII, 2BII and 2CII represented the dynamic trend of the steady-state activity of monophenolase with various concentrations of the inhibitors. The concentration of Y_1_, Y_2_ and Y_3_ to decrease half of the steady-state activities of monophenolase was 185, 245 and 95 μM, respectively. The results showed that the inhibitory effects of the compounds on the reaction rates of diphenolase and monophenolase were dose-dependent. A conclusion could be drawnj from Fig 2 that inhibitory effects of inhibitors on diphenolase were higher than that of monophenolase inhibition.

### Inhibitory mechanisms, types, and constants of the compounds on mushroom tyrosinase

**Fig 3:**
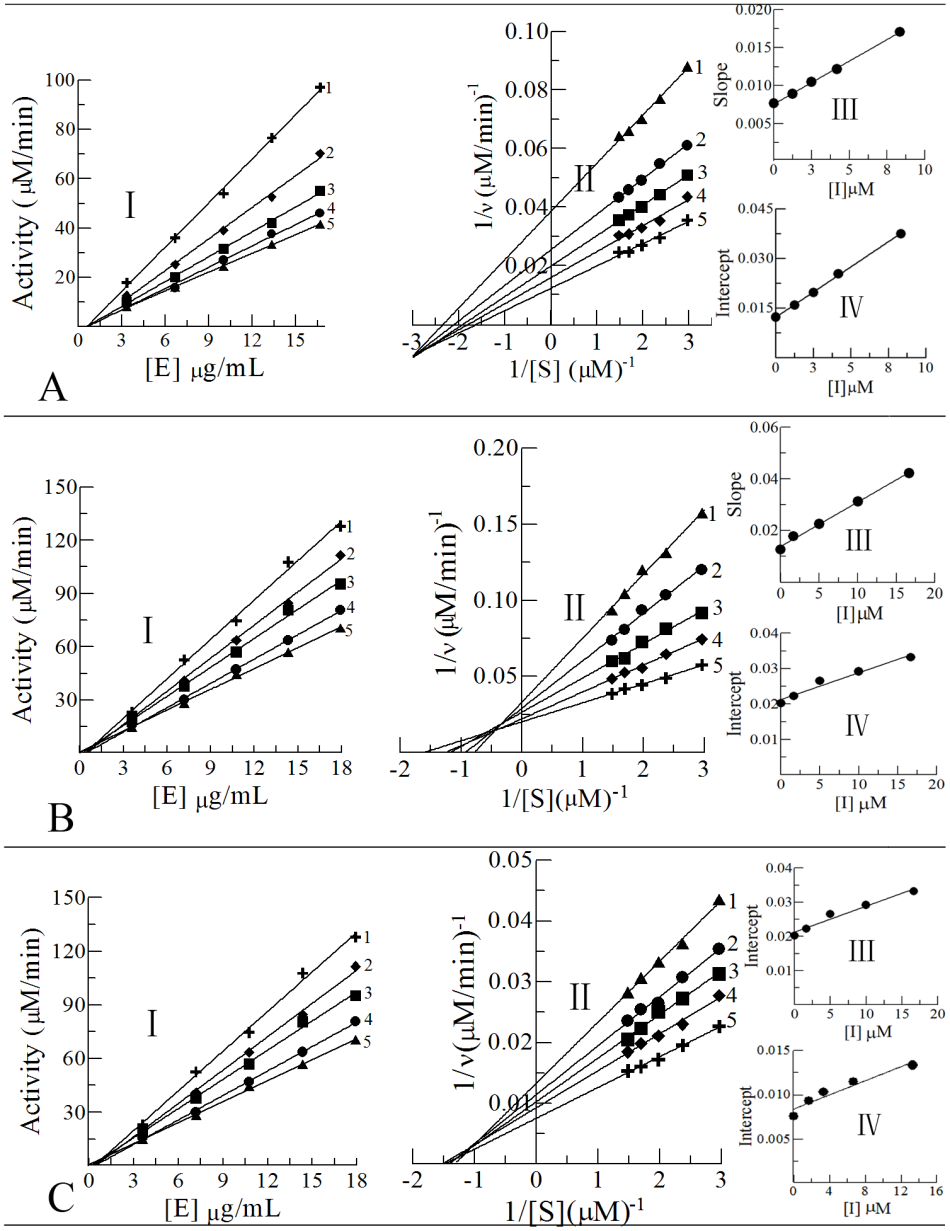
Determinations of the inhibitory mechanisms, types, and constants on diphenolase. (**I**) The inhibitory mechanisms of the compounds Y_1_ to Y_3_. (**II**) Lineweaver-Burk plots for diphenolase activity. (**III**) The plots of slopes versus the concentrations of the compounds (**IV**) The plots of intercepts versus the concentrations of the compounds.

Under different concentrations of the enzyme, the residual activity of tyrosinase with a fixed amount of substrate was tested. The results were showed in Fig 3AI, 3BI and 3CI. The straight lines 1 to 5 all passed through the origin, which indicated that the inhibitory mechanisms of Y_1_, Y_2_ and Y_3_ on tyrosinase activity were reversible.

In order to explore the inhibitory types of the Schiff's bases Y_1_ to Y_3_, the concentration of tyrosinase was kept constant and measured the initial reactions velocity (v_0_) under various concentrations of substrate were measured. A set of straight lines (Fig 3AII, 3BII and 3CII) of 1/*v*_0_ versus 1/[S] all passed through the second quadrant or third quadrant. The results indicated that the inhibitory types between the inhibitors and tyrosinase were of mixed type including competitive, non-competitive and anti-competitive inhibition. The constants K_I_, for the inhibitor bonding with the free enzymes, were shown in Fig 3AIII, 3BIII and 3CIII. Furthermore, the constants K_IS_, for the inhibitors bonding with enzyme-substrate complexes, were shown in Fig 3AIV, 3BIV and 3CIV. K_I_ and K_IS_ were acquired from the straight lines of the slopes and vertical intercepts versus the concentrations of inhibitors, respectively.

KI and K_IS_ Its values of Y_1_, Y_2_ and Y_3_ were determined to be 6.67 and 4 μM, 7.94 and 27.8 μM, 15.47 and 21.04 μM, respectively. The parameters of inhibition were summarized in Table 1.

**Table 1:** The Inhibition parameters of compounds Y_1_-Y_3_ on tyrosianse.

parameters	Y_1_	Y_2_	Y_3_
*IC*_50_(μM)	12.5	7	1.52
*K*_I_	6.67	7.49	15.47
*K*_IS_	4	28	21.04
Inhibition type	Mixed	Mixed	Mixed
Inhibitory mechanisms	Reversible	Reversible	Reversible

### Fluorescence quenching

Fluorescence quenching can be divided into static quenching and dynamic quenching. Dynamic quenching is a process, which has energy or electron transfer, while the static quenching process will generate complexes without fluorescence [42]. The quenching rate between the fluorescent molecules and quenching agents followed the Stem.Volmer curve equation [43]:

$$F_{0}/F=1+K_{SV}\left[Q\right]$$

**Fig 4:**
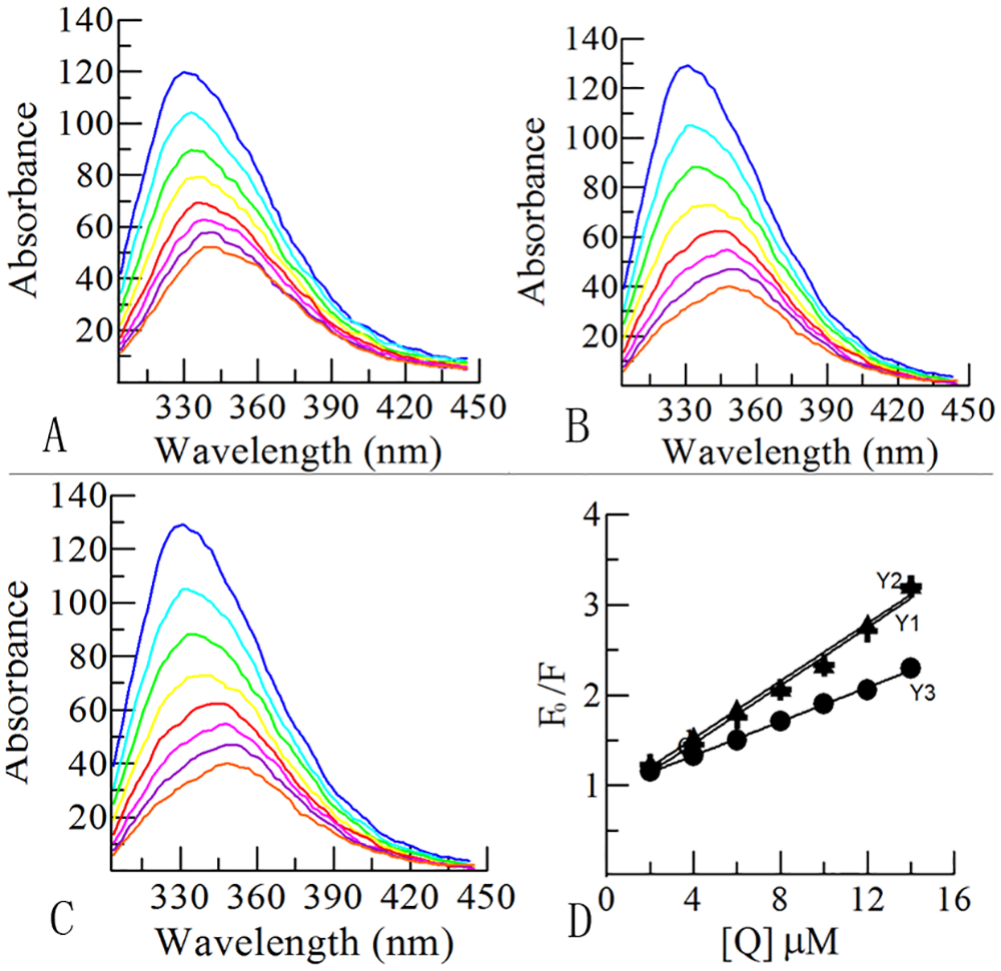
The fluorescence quenching experiment of compounds. **(A, B, C)** Fluorescence absorption phenomena of the compounds. A, B and C represents the compound Y_1_, Y_2_ and Y_3_ respectively. (**D**) The relationship of fluorescence intensity and the concentrations of compounds Y_1_, Y_2_ and Y_3_.

In the formula, F_0_ is the intensity of fluorescence without quenching agent, F is the fluorescence intensity after adding quencher, K_sv_ (L/mol) represents the relationship of dynamic equilibrium between biological macromolecules and fluorescence quencher molecules after diffusing and colliding, and [Q] is the concentration of quencher. From Fig 4D, the K_sv_ of Y_1_, Y_2_ and Y_3_ was determined to be 94437, 7160983 and 159186 L/mol, respectively. The rate constants of K_s_v < 100 L/mol in dynamic quenching process indicated that the quenching process was not controlled by diffusion but perhaps a static quenching process which will have an impact on protein secondary structure and physiological activity.

In addition, the fluorescence intensity decreased and the emission wavelength values slightly increased with the concentration of inhibitors increasing, which further illustrated the generation of complexes between inhibitors and tyrosinase (Fig 4A, 4B and 4C).

### Copper interaction

**Fig 5:**
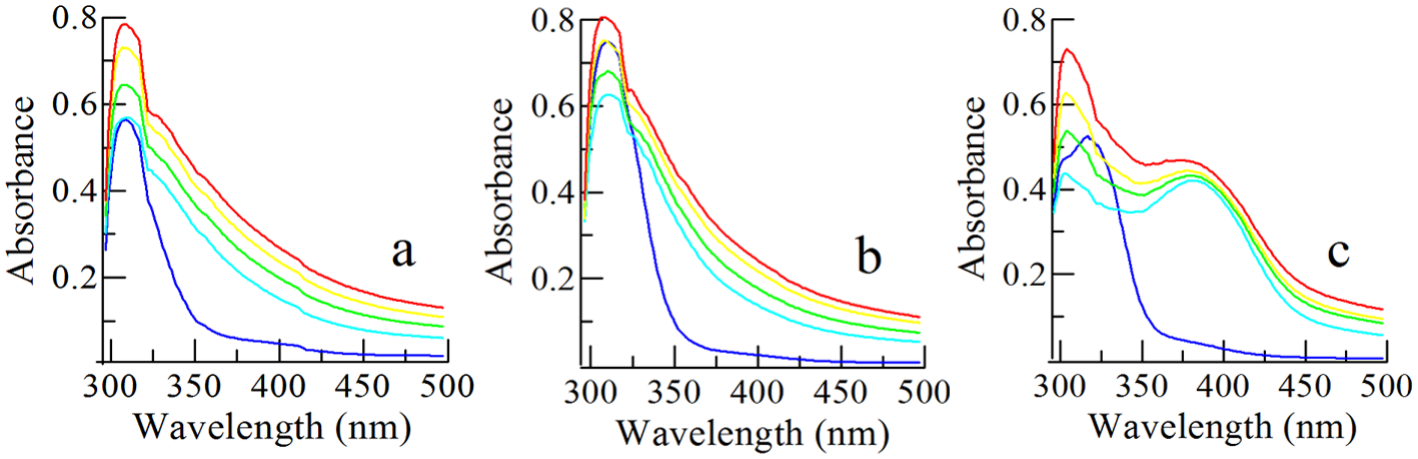
Absorption spectra for the copper ions interaction with the compounds Y_1_, Y_2_ and Y_3_, respectively.

Tyrosinase has two copper ions in its active center, which catalyzes the adjacent hydroxylation of monophenol into diphenol and then catalyzes the reaction of diphenol into quinones [44]. The whole wavelength scanning experiment detected the bonding ability of the three compounds and copper ions, which was used to infer the intensity of the inhibitors to combine with tyrosinase and reveal inhibitory mechanisms. The results were shown in Fig 5.

As observed in Fig 5a, 5b and 5c, a new absorption peak was generated at about 380 nm in Fig 5c, thus only compound Y_3_ could combine with copper ion. A conclusion could be speculated: Y_3_ could combine with the copper ions in the active center of tyrosinase and the ability of the substituted group to chelate the copper ions was related to its location on the benzene ring. And the hypothesis will be further confirmed by molecular docking analysis.

### Antioxidant assay

**Fig 6:**
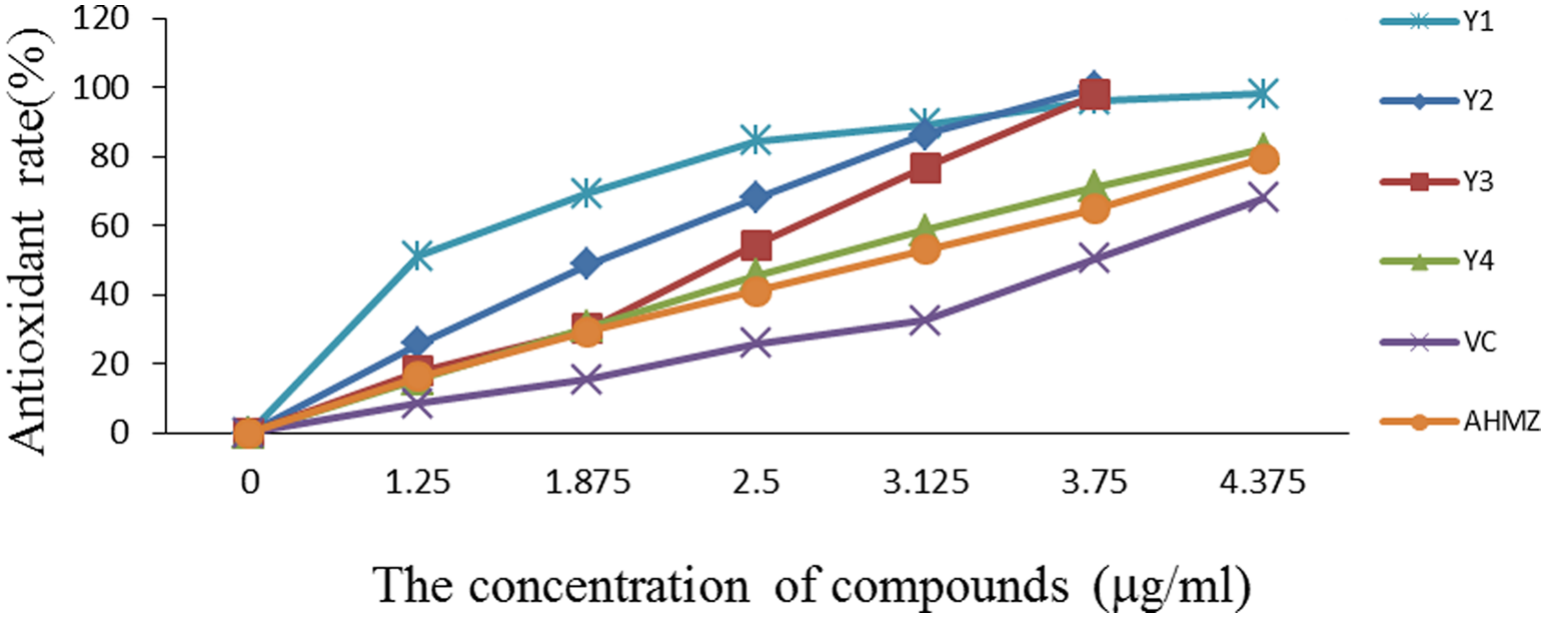
The antioxidant assays of compounds Y_1_ to Y_4_.

Triazole derivatives generally have extensive biological activities, because of its thiol and hydroxyl groups, it may have strong antioxidant activity. With vitamin C (Vc) and AHMZ as control, the antioxidation abilities of the compounds Y_1_ to Y_4_ were assayed and the results were shown in Fig 6.

The determination of antioxidant activity achieved the desired results and the properties were dose-dependent. The antioxidant capacities were in the range of Y_1_ > Y_2_ > Y_3_ > Y_4_ > Vc > AHMZ. It can be concluded that the combination of A ATMZ and benzaldehyde could improve the antioxidant capacity. This result may be explained by the superimposition effect of reducing groups (hydroxyl, thiol and amino).

### Molecular docking

**Fig 7:**
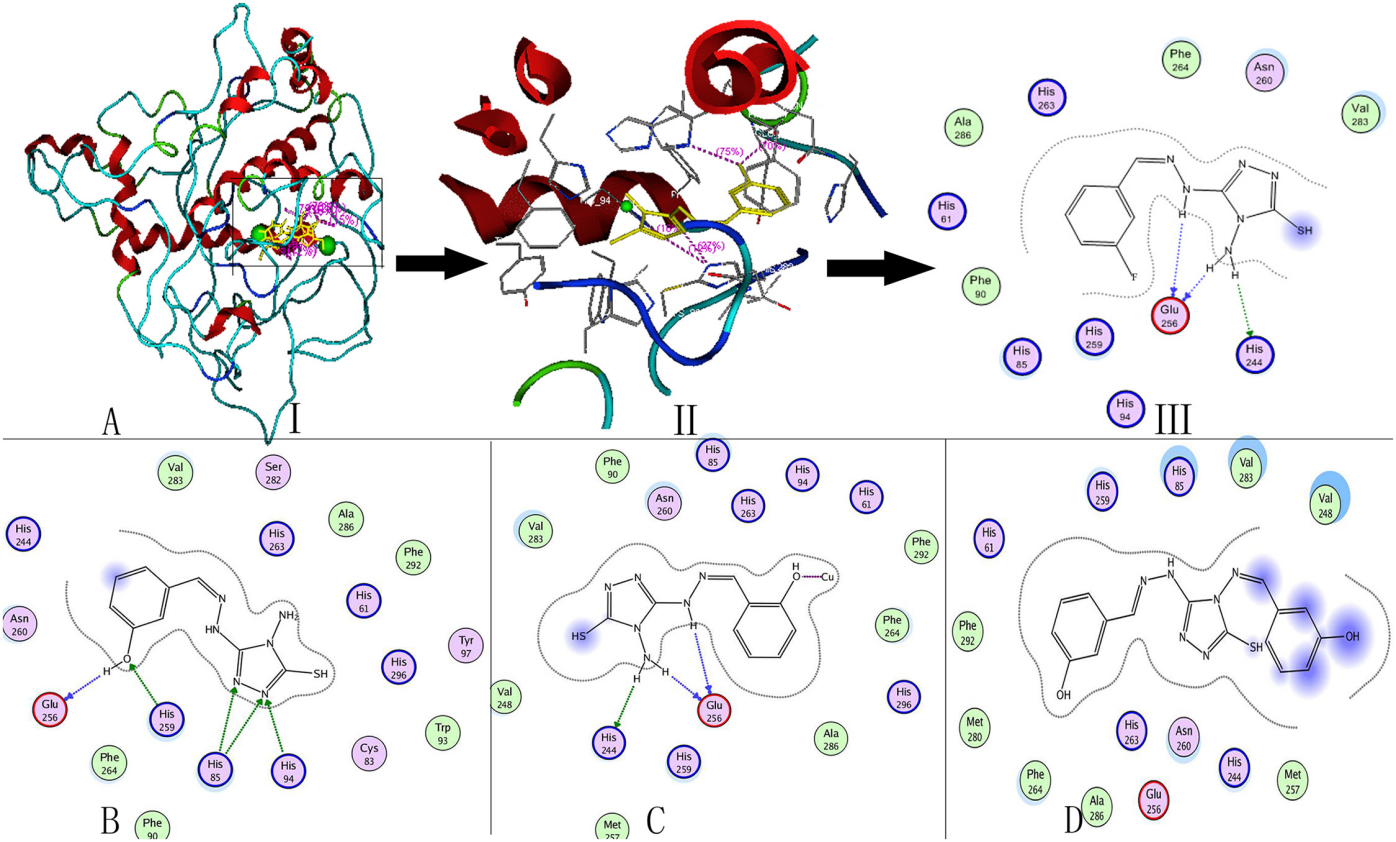
Docking information of compound Y_1_ to Y_4_ in the bonding site of tyrosinase. (**A**) The molecular docking process of compound Y_1_. (**I**)The global figure of docking. (**II**)The enlarged figure of docking center. The inhibitor molecule was shown as yellow, while the copper ion was in green. (**III, B, C, D**) the interaction between compound Y_1_ to Y_4_ and the residues of tyrosinase, respectively.

Molecular simulations further clarified the underlying mechanisms of compounds in the active center, which would give a more convincing conclusion by combining the results of copper ions mutual effect. Fig 7 depicted the docking conformation of the four compounds in the tyrosinase catalytic center.

For the substituent on the benzene ring, only the hydroxyl of compound Y_3_ could interact with the copper ion in the active center of enzyme. Some atoms on compound Y_1_ to Y_3_ act as the acceptor or donor generate hydrogen bonds with the center of tyrosinase. Compound Y_1_ generated hydrogen bonds with His_244_ and Glu_256_, Y_2_ with Glu_256_, His_259_, His_85_, His_94_, Y_3_ with His_244_ and Glu_256_. In addition compound Y_1_ could also form interactions with tyrosinase residues: Ala_286_, His_263_, Phe_264_, Asn_260_, Val_283_, His_94_, His_259_, His_85_, Phe_90_, His_61_, which was shown in Fig 7AIII. Compound Y_2_ formed interactions with tyrosinase residues: Val_283_, Ser_282_, His_263_, Ala_286_, Phe_292_, His_61_, His_296_, Tyr_97_, Trp_93_, Cys_83_, Phe_90_, Phe_264_, Asn_260_, His_244_, which was shown in Fig 7B. Compound Y_3_ form interactions with tyrosinase residues: Val_283_, Phe_90_, Asn_260_, His_85_, His_263_, His_94_, His_61_, Phe_292_, Phe_264_, His_296_, Ala_286_, His_259_, Met_257_, with the result was shown in Fig 7C. Moreover, compound Y_4_ could also form interaction with tyrosinase residues: Val_248_, Val_283_, His_85_, His_259_, His_61_, Phe_292_, Met_280_, Phe_264_, Ala_286_, His_263_, Asn_260_, His_244_, Met_257_, Glu_256_, which was shown in Fig 7D.

## Discussion

The methods of decreasing the activity of melanocytes or avoiding UV radiation all could effectively reduce the accumulation of melanin. However, inhibiting the activities of tyrosinase was most effective when the damage has been done [45,46]. For the purpose of acquiring efficient antityrosinase agents, we synthesized 10 compounds and 3 from them were found to exhibit antityrosinae activities. This article reported the synthesis of tyrosinase inhibitors and screening on monophenolase and diphenolase of tyrosinase, tested the inhibitory types, mechanisms, fluorescence quenching, copper ions interaction, molecular simulation to further unravel the mechanisms, and its oxidation resistance as a supplement of biological activity.

Compounds Y_1_ to Y_3_ have inhibitory effect on monophenolase and diphenolase activities of mushroom tyrosinase, and the inhibition effects on enzyme were dose-dependent. For monophenolase activity, the compounds suppressed the steady-state activities drastically to decrease the process of enzyme catalysis. For the diphenolase activity, reversible inhibition indicated that the inhibitors suppressed tyrosinase not through reducing the number of the dynamic enzymes. The mixed inhibitory types showed that Y_1_, Y_2_ and Y_3_ suppressed the enzyme activities through bonding with the free enzymes and the enzyme-substrate complexes. Fluorescence quenching results reflected the tyrosinase molecular conformation could be changed by the quencher, which directly showed that tyrosinase could combine with inhibitors to form. The result revealed that the inhibition on tyrosinase did not include the anti-competitive inhibition and the bonding between inhibitors and enzyme may alter the conformations of bonding sites of the substrate to make the bonding difficult or the inhibitor may combine with enzyme-substrate complex and changed the conformation of enzyme to form the inactive inhibitor-enzyme-substrate complexes. Copper ions interaction and molecular docking experiment further exposed the reason of the inhibitory effect on tyrosinase. The inhibition abilitise of the inhibitors on tyrosinase were closely related to the structures of the inhibitors. From the slight difference of molecular structures in the docking information, the following information could be get: the free amino hydrogen on imidazole ring had the potential ability to inhibit the activity of tyrosinase by forming hydrogen bonds with residues of the enzyme active center. The molecular structures of compounds Y_1_ to Y_4_ proved that the inhibitory effects of 2-substitutes on the benzene ring were better than 3-substitutes and hydroxyl substitutes were better than that of fluorine substitutes. In addition, two free amino groups on the imidazole ring connected with two benzene rings respectively, the groups would generate greater steric hindrance and affect the bonding between tyrosinase and inhibitors resulting in reduced inhibitory effect. The differences of structure affect the degree of inhibition on the enzyme and the way of interaction with the enzyme which is in agreement with previous reports [47,48].

The antioxidant abilities of the compounds Y_1_ to Y_4_ as a supplement of biological activity were assayed. Comparing the differences of molecular structures, the position and type of the substituent on the benzene ring affected the antioxidant capacity. The results that fluorine substitutes were superior to hydroxyl substitutes, and that 3-substituted on the benzene ring were better than 2-substitutes were in contrast to their antityrosinase effects. The antioxidant mechanisms of the compounds are different form their antityrosinase mechanisms, which needs further studies.

In summary, three novel compounds were synthesized as antityrosinase agents. Through many primary determinations, the inhibitors showed effective inhibitory effects on mushroom tyrosinase and the inhibition mechanisms were unraveled. Our current work offered some guidance to the design of novel tyrosinase inhibitors. However, further studies are needed to explore their inhibitory effect on excessive synthesis of melanin, their usage in fresh-keeping, or their other biological activities.

## Supporting Information

S1 Fig The IR spectrum of Compound Y_1_. (TIF)

S2 Fig The ^1^H NMR spectrum of Compound Y_1_. (TIF)

S3 Fig The LC-MS spectrum of Compound Y_1_. (TIF)

S4 Fig The IR spectrum of Compound Y_2_. (TIF)

S5 Fig The ^1^H NMR spectrum of Compound Y_2_. (TIF)

S6 Fig The LC-MS spectrum of Compound Y_2_. (TIF)

S7 Fig The IR spectrum of Compound Y_3_. (TIF)

S8 Fig The ^1^H NMR spectrum of Compound Y_3_. (TIF)

S9 Fig The LC-MS spectrum of Compound Y_3_. (TIF)

S10 Fig The IR spectrum of Compound Y_4_. (TIF)

S11 Fig The ^1^H NMR spectrum of Compound Y_4_. (TIF)

S12 Fig The LC-MS spectrum of Compound Y_4_. (TIF)

S13 Fig The article summary. Synthesis of triazole schiff’s base derivatives and the study of tyrosinase inhibitory mechanism.
(TIF)
