# Supplementary figures and images for: Synthesis of Triazole Schiff’s Base Derivatives and Their Inhibitory Kinetics on Tyrosinase Activity
Source: PLoS One. 2015 Sep 30;10(9):e0138578. doi: 10.1371/journal.pone.0138578 (PMC4589318; doi:10.1371/journal.pone.0138578)

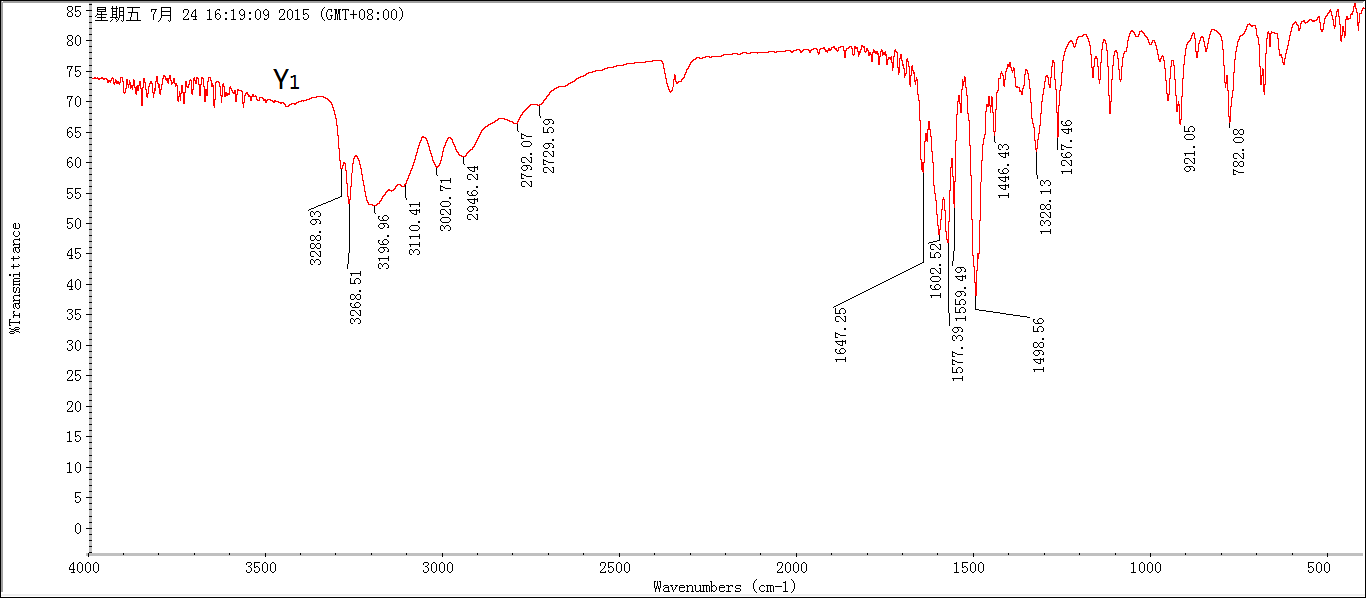

Supplement: S1 Fig — (TIF) [file pone.0138578.s001.TIF]

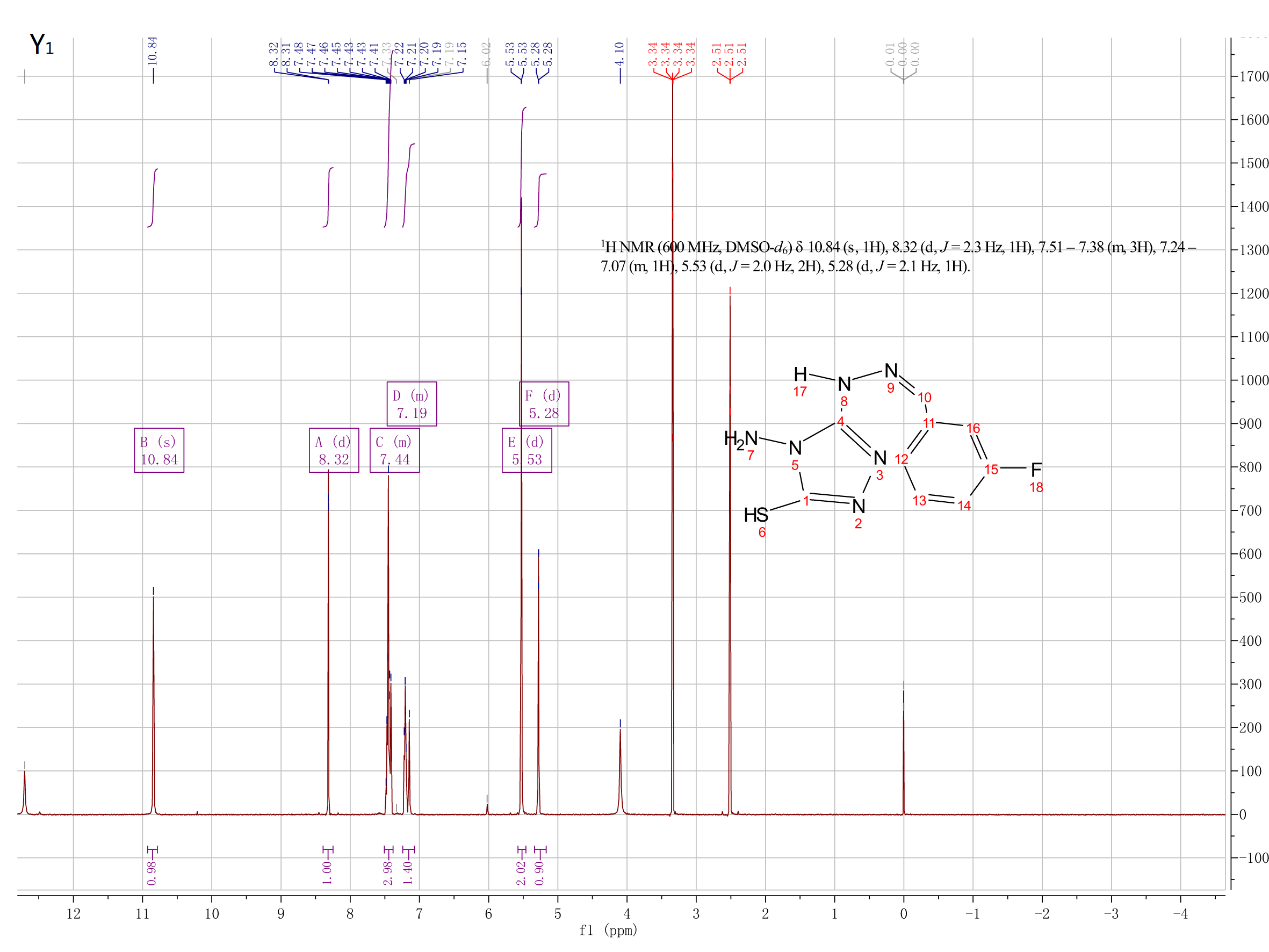

Supplement: S2 Fig — (TIF) [file pone.0138578.s002.tif]

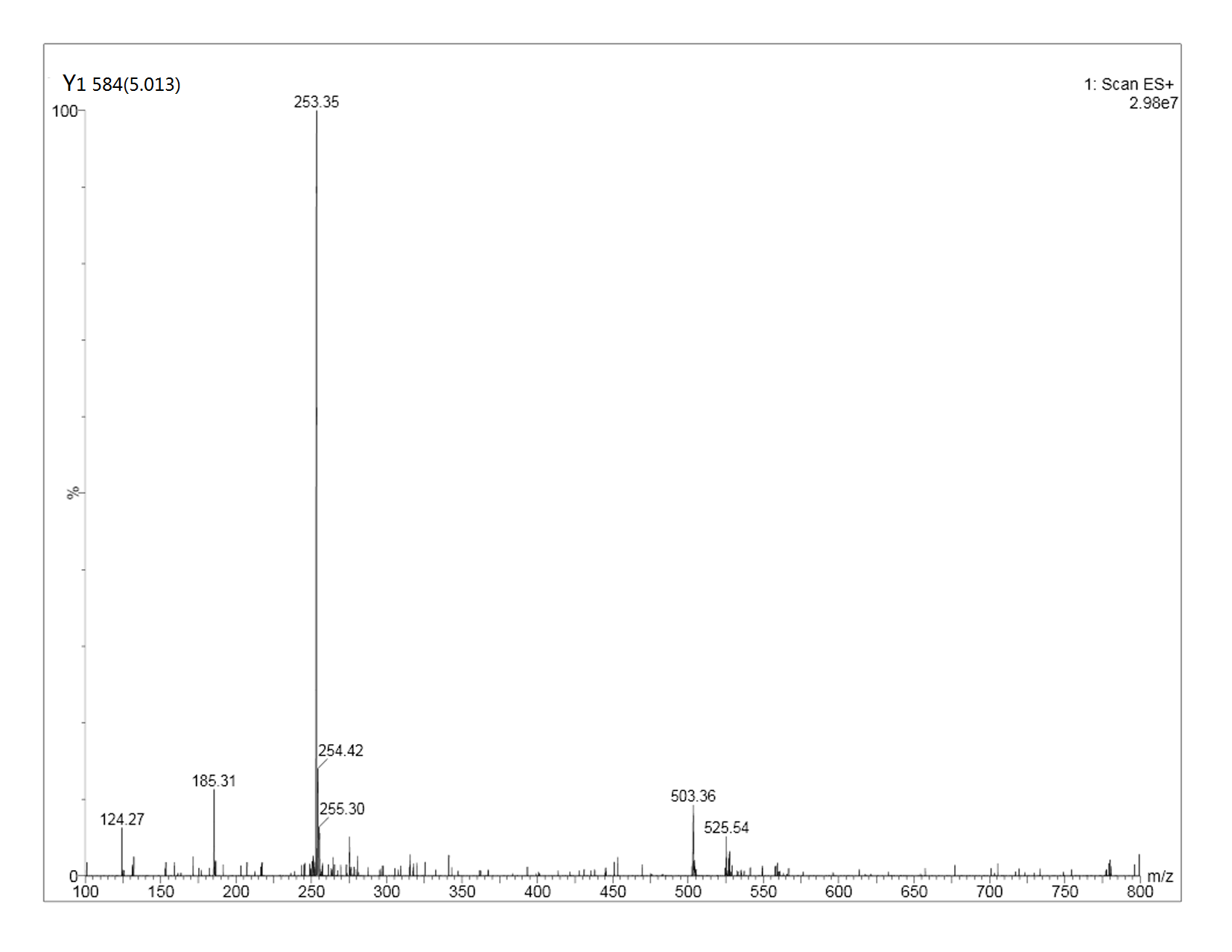

Supplement: S3 Fig — (TIF) [file pone.0138578.s003.tif]

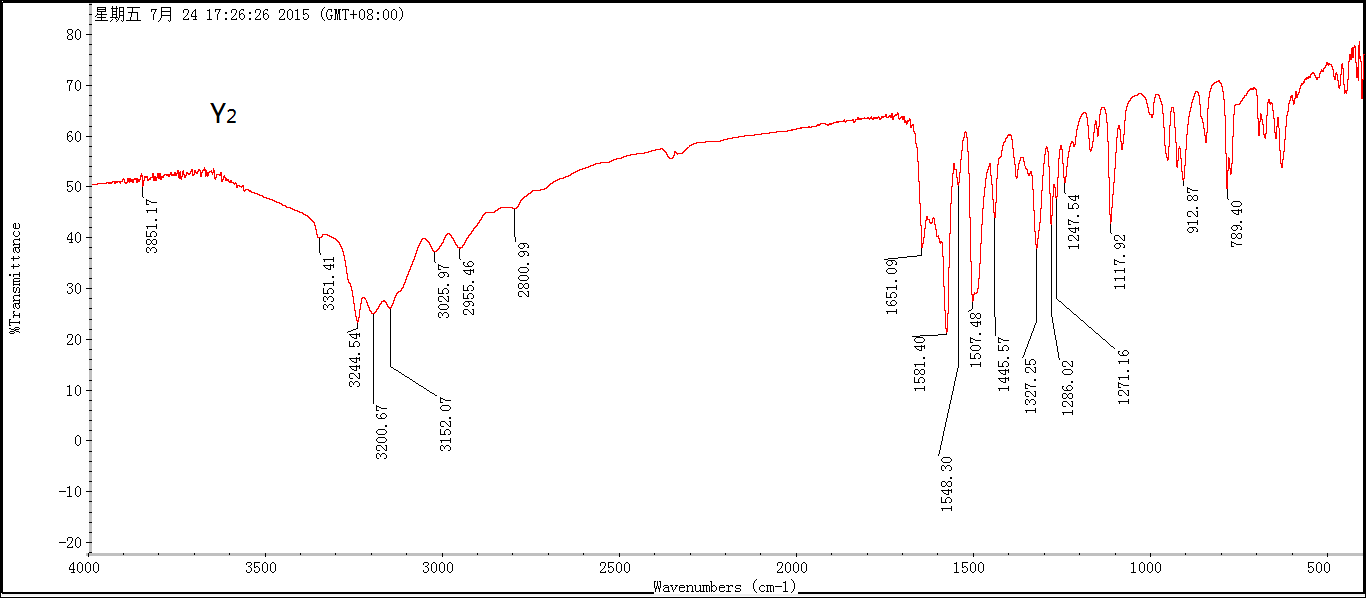

Supplement: S4 Fig — (TIF) [file pone.0138578.s004.TIF]

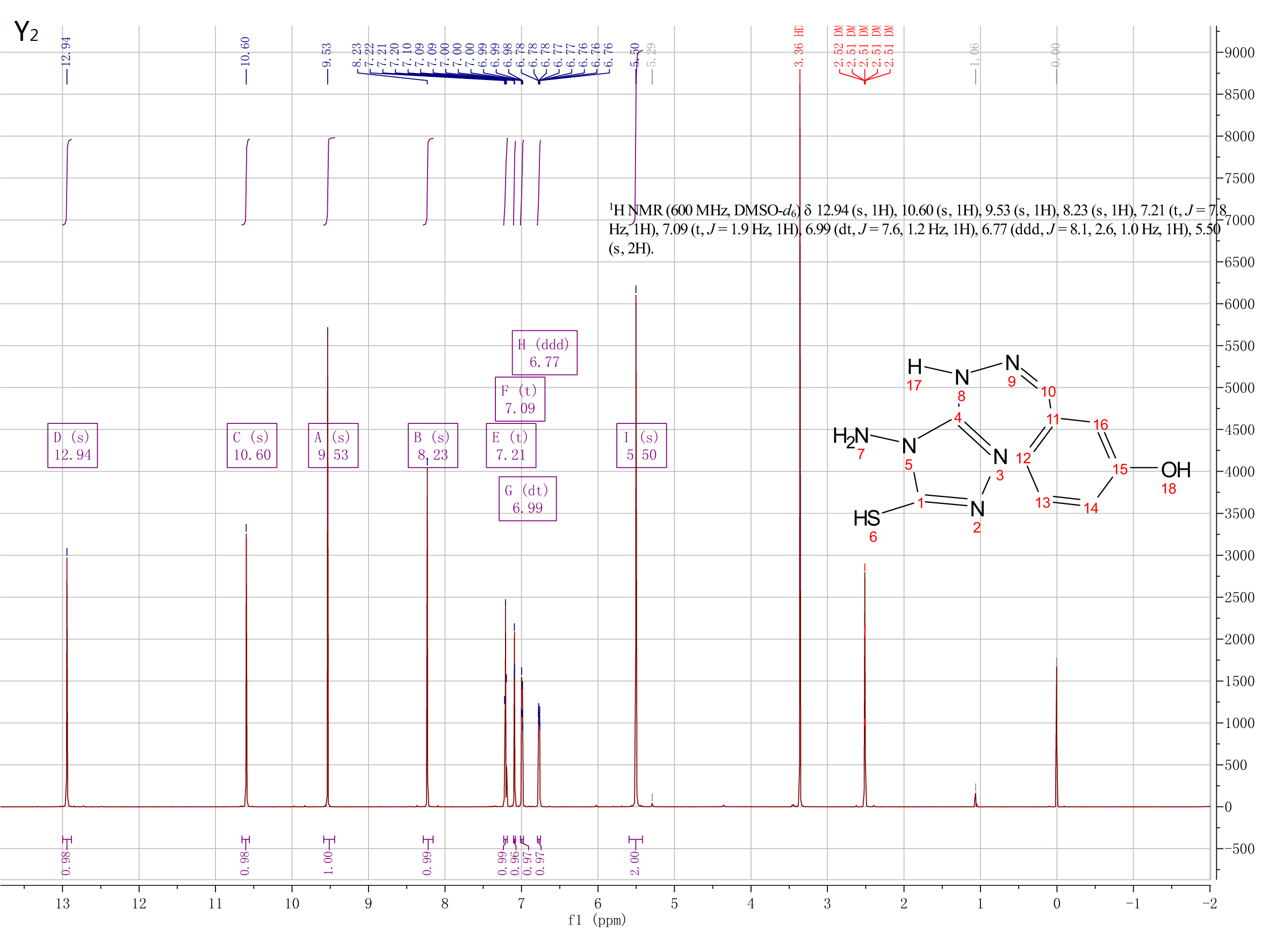

Supplement: S5 Fig — (TIF) [file pone.0138578.s005.tif]

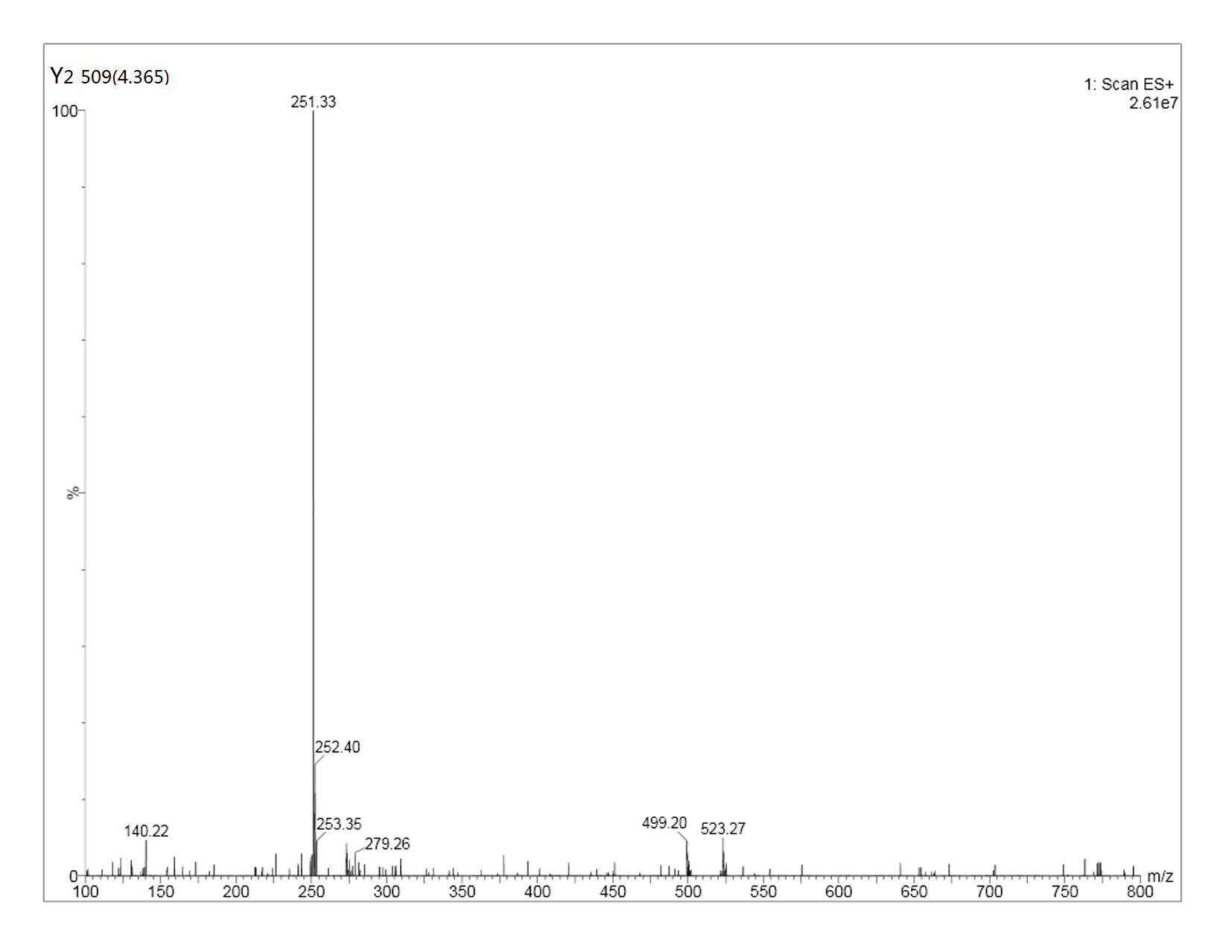

Supplement: S6 Fig — (TIF) [file pone.0138578.s006.tif]

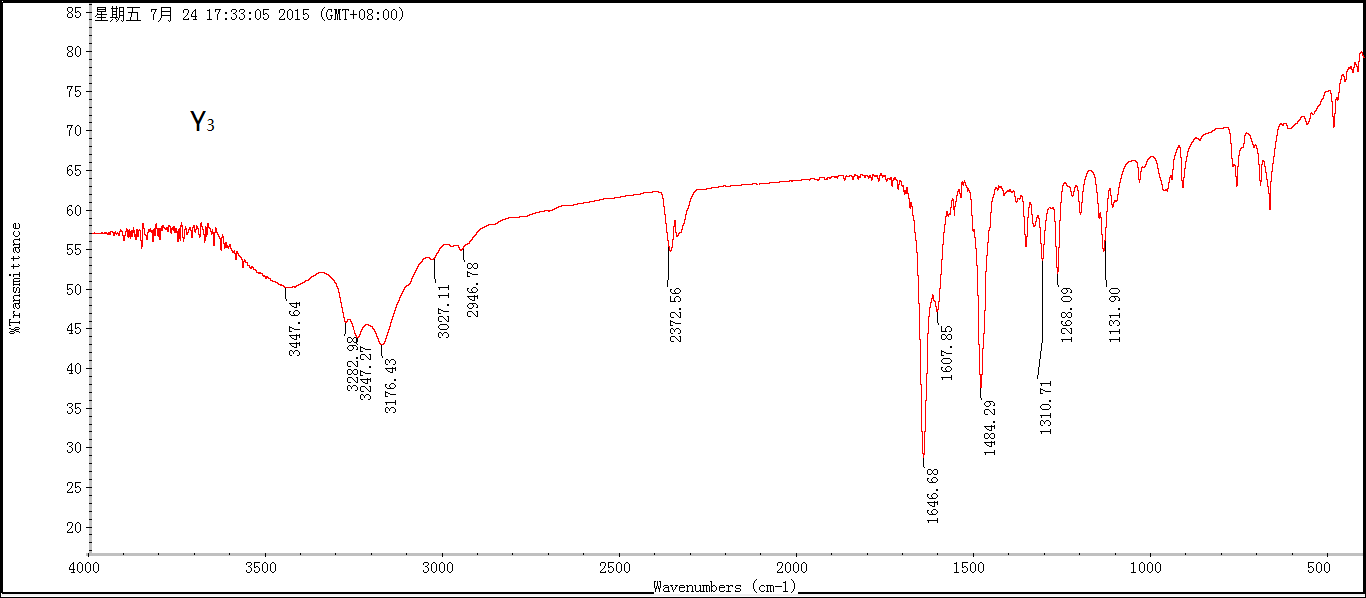

Supplement: S7 Fig — (TIF) [file pone.0138578.s007.TIF]

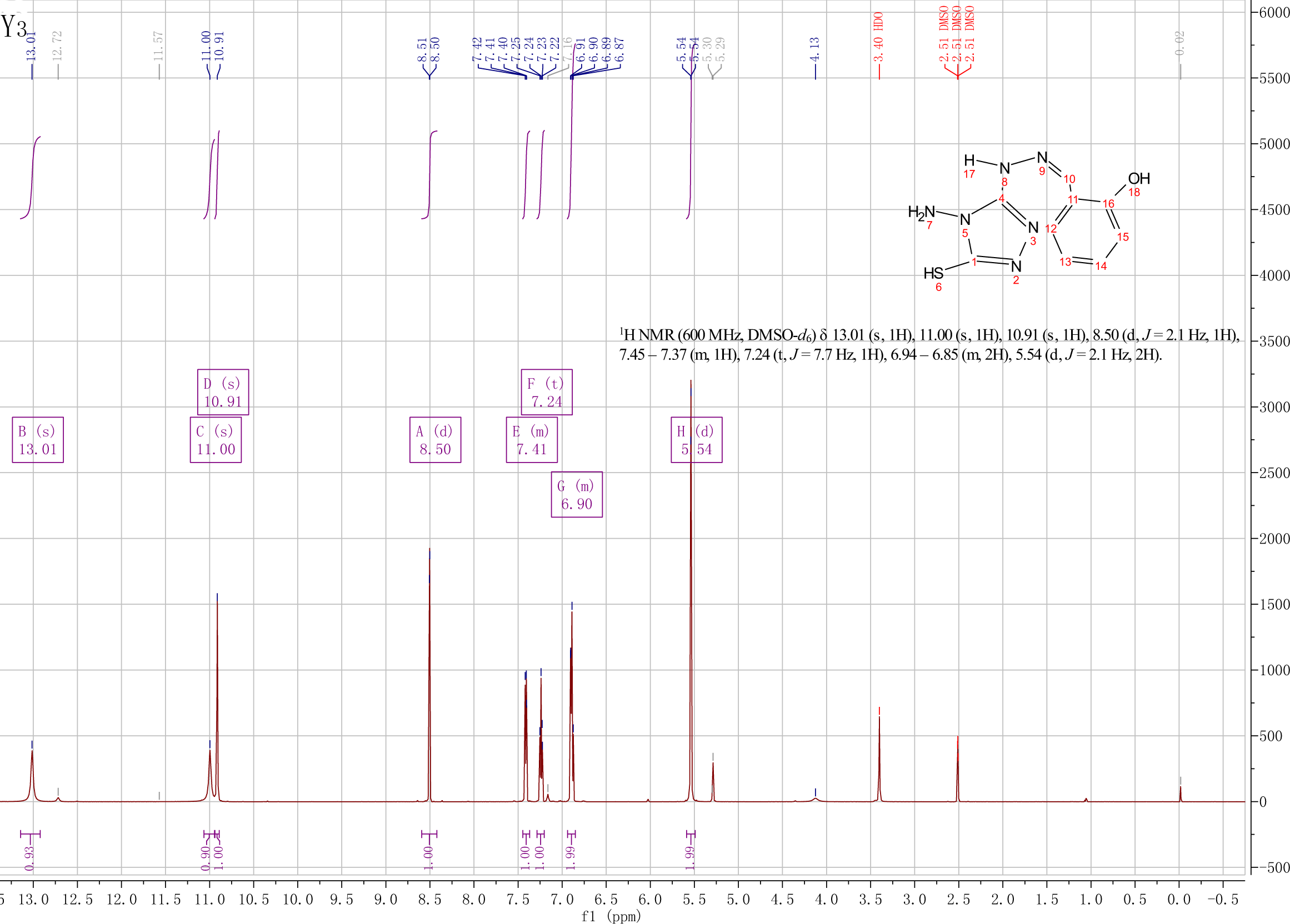

Supplement: S8 Fig — (TIF) [file pone.0138578.s008.tif]

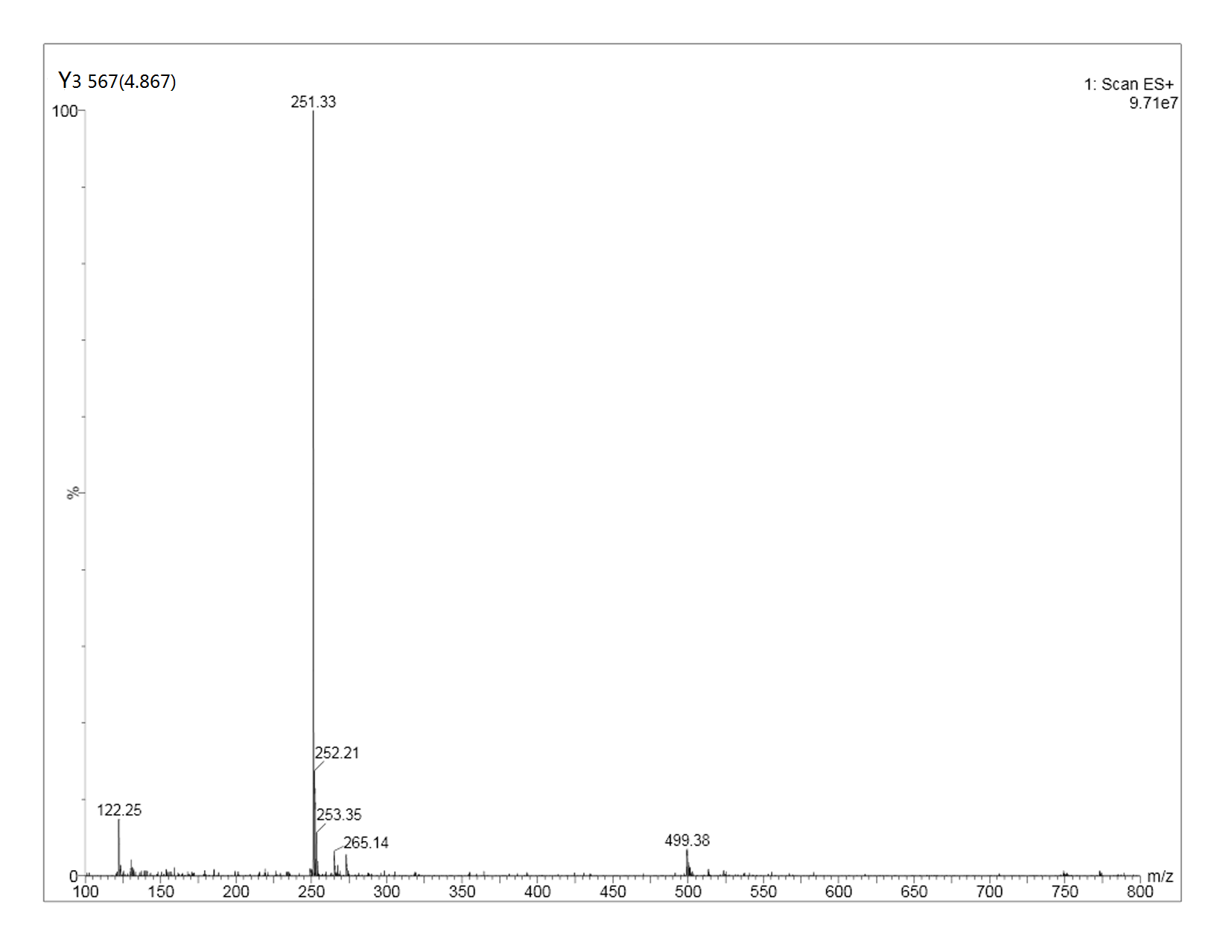

Supplement: S9 Fig — (TIF) [file pone.0138578.s009.tif]

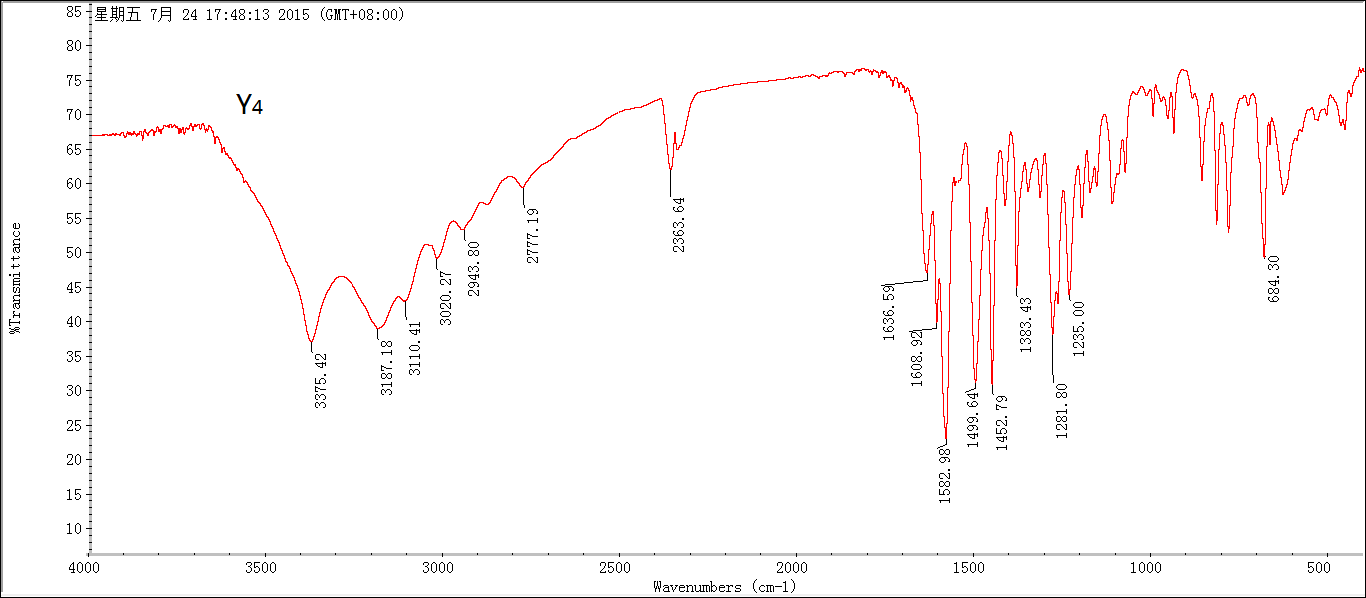

Supplement: S10 Fig — (TIF) [file pone.0138578.s010.TIF]

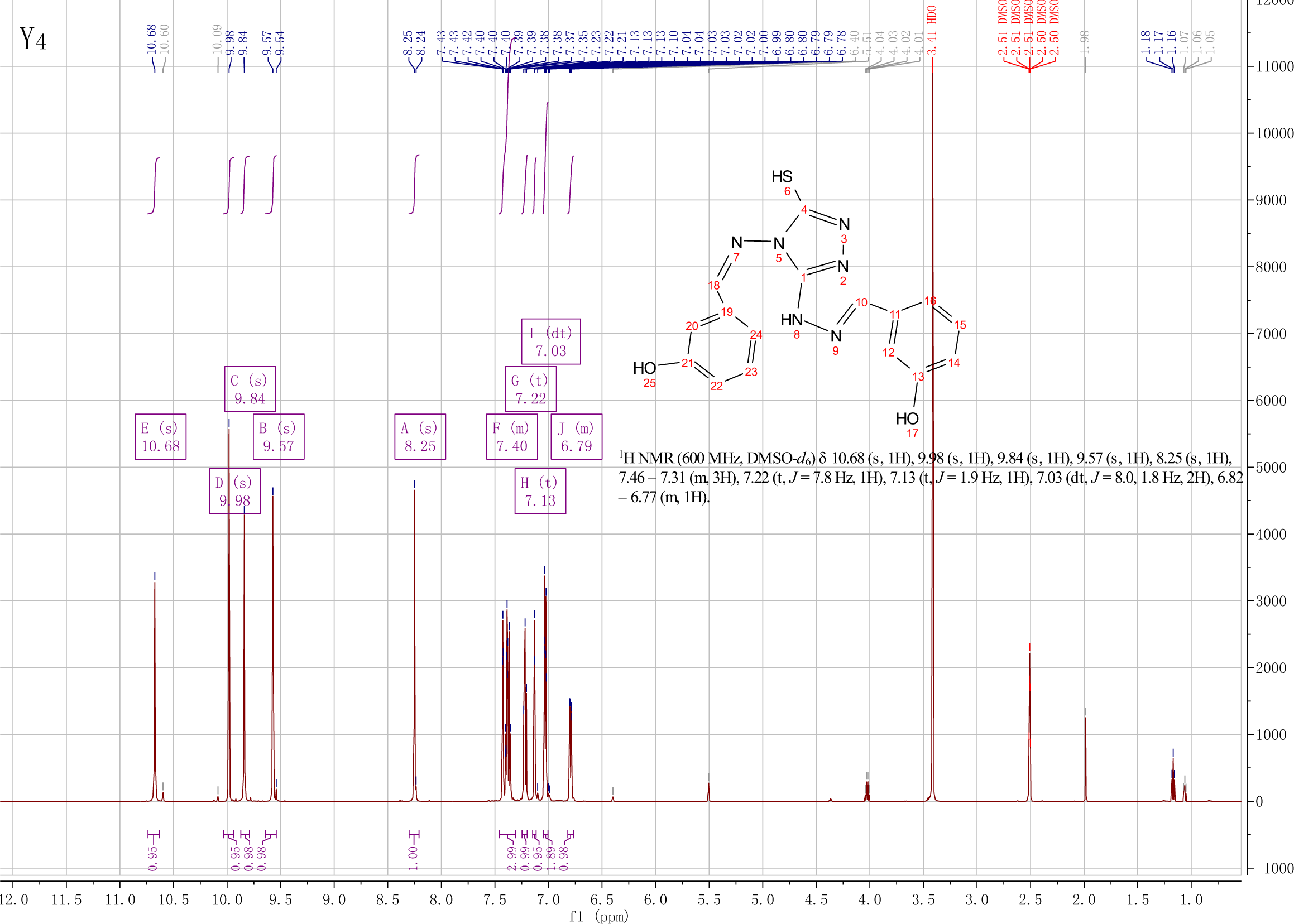

Supplement: S11 Fig — (TIF) [file pone.0138578.s011.tif]

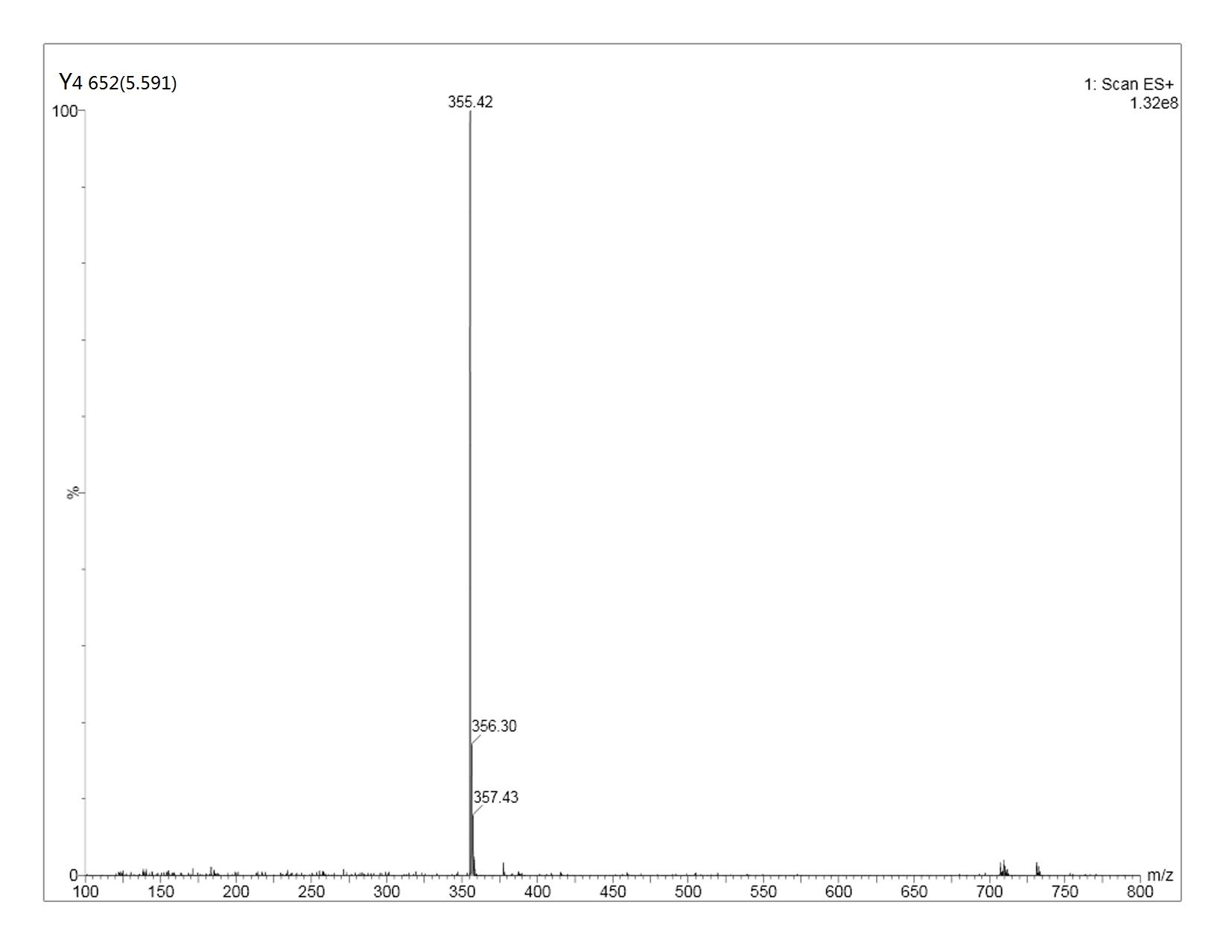

Supplement: S12 Fig — (TIF) [file pone.0138578.s012.tif]

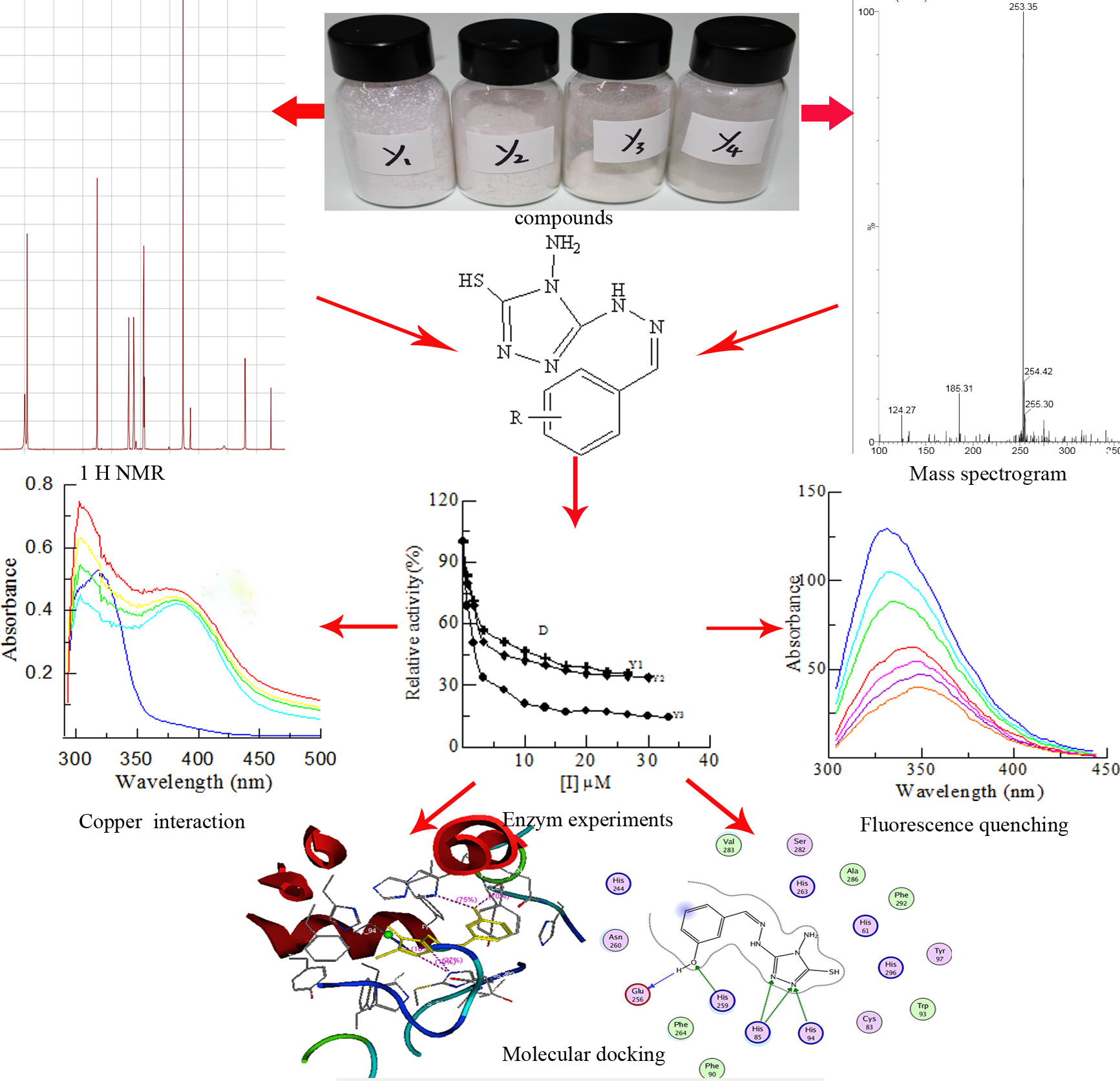

Supplement: S13 Fig — Synthesis of triazole schiff’s base derivatives and the study of tyrosinase inhibitory mechanism. (TIF) [file pone.0138578.s013.tif]
